# Supplementary material for: Role of informal healthcare providers in tuberculosis care in low- and middle-income countries: A systematic scoping review
Source: PLoS One. 2021 Sep 2;16(9):e0256795. doi: 10.1371/journal.pone.0256795 (PMC8412253; doi:10.1371/journal.pone.0256795)
Supplement: S2 File — (PDF) [file pone.0256795.s002.pdf]

Table 1: Definitions of Informal Healthcare Providers in previous publications

S. N	Topics	Terminologies used	Definitions	Type of research	Study area	Publication venue
1	Informal sector providers in Bangladesh: how equipped are they to provide rational health care?	Informal health care providers	The healthcare providers who are not registered with any government regulatory body.	Nationwide health-care provider survey	Bangladesh	Health Policy and Planning
2	Making health markets work better for poor people: the case of informal providers.	Informal providers	The term 'informal' includes a great variety of providers who routinely or occasionally undertake activities for which they do not possess the required medical certification, as assessed by health authorities and/or legislation covering the provision of health services.	Review of two studies	Bangladesh and Nigeria	Health Policy and Planning
3	Perceptions of appropriate treatment among the informal allopathic providers: insights from a qualitative study in two peri-urban areas in Bangladesh	Informal allopathic providers	In Bangladesh, the term refers to those who are not registered with any government regulatory body, therefore operate beyond government's oversight. Generally, they do not receive any training from any recognized medical training institution; however, workshop, seminar, and apprenticeships are their typical learning platforms. Informal providers' practices are usually small and localized, and dependent on the maintenance of good networking and relationships with the communities.	Exploratory study	Bangladesh	BMC Health Services Research
4	Integrating informal providers into a people-centered health systems approach: qualitative evidence from local health systems in rural Nigeria	Informal providers	Informal healthcare providers encompass a wide range of practitioners who provide services for which they do not have formal medical training or that are outside the boundaries of their licensure.	Qualitative study	Nigeria	BMC Health Services Research

5	What Is the Role of Informal Healthcare Providers in Developing Countries? A Systematic Review	Informal healthcare providers	IPs must meet our first criteria below, and at least two of the remaining three criterion. The set of definitional criteria include: 1.**Training**: IPs include those who have not received formally recognized training with a defined curriculum from an institution (i.e. government, NGO, or academic institution).IPs, however, typically have some level of informal training through apprenticeships, seminars, and workshops, and are typically not mandated by any formal institution.2.**Payment**: IPs collect payment from patients served, not from institutions. One notable exception to this criterion involves NGO or other sponsored voucher programs, where informal providers exchange services or goods for payment from a sponsoring body in the form of reimbursement vouchers. Payment is usually, but not always, un-documented and tendered in cash. IPs are chiefly entrepreneurs.3.**Registration and regulation**: IPs are not typically registered with any government regulatory body and operate outside of the purview of regulation, registration, or oversight by the government or other institutions.4.**Professional affiliation**: IP professional associations, if they exist, are primarily focused on networking and business activities and conduct minimal self-regulation. Because caregivers do not receive payment for services, and therefore fail to meet the first of our key definitional criteria, we excluded them even though they feature many of the same traits and practice in a similar fashion to other IPs. Community health workers who were trained by NGOs or governments are not included in the study. Only studies that specify ''untrained'' community health workers are included the study results.	Systematic Review	Developing countries	PLOS One
6	Can interventions improve health services from informal private providers in low and middle-income countries?: a comprehensive review of the literature.	Informal private provider	We have chosen to define IPPs as those who provide allopathic medical treatment or services to the public but have not received formal training in allopathic medicine. Providers who do meet our criteria include drug compounders working as village doctors, traditional healers who advise clients to take antibiotic remedies, health care entities who operate under a business license but without a health-related certification or accreditation, and pharmacists who diagnose and treat patients, thus practicing beyond their level of training. Our definition also includes lay or community health workers who have been working independently in the health market, or if they retain their independence to work in the market when they are part of a government or NGO programme. Volunteers are excluded from our study since we assume that they are not engaged in	Review of literature	LMICs	Health Policy and Planning

			market transactions. Cadres of health workers that are created as part of a research project and are entirely dependent on a research project for their support are not included in this review, because the projects have not been designed for the health worker to continue working in the market. Because of our interest in the informal sector, we do not include interventions where community health workers were trained and employed solely for the research initiative or where they were formally trained and registered through a government agency.			
7	Engaging Informal Providers in TB control: What Is the Potential in the Implementation of the WHO Stop TB Strategy? A Discussion Paper	Informal Providers	Informal providers are defined here as individuals working independently (not accountable to any institution or organization), providing health-related services in a context not formally structured or clearly regulated (e.g., practitioners of traditional medicine in African countries); or practitioners not fully qualified to provide the type of services they offer (e.g., non-qualified providers, village doctors, drug shops, village injectionists, etc.).	Discussion paper	Globally	World Health and Population

As to the rigour of the definition, we reviewed the definitions in other publications. We felt a need for a more flexible and broader approach considering the nature of the review. There were many considerations in the definition we used in our review, and they were:

1. IPs were not registered or affiliated with a government or a recognized non-governmental organization. We expanded our definition to include private institutions, not limiting it to the government system as in previous studies. Some Community Health Workers (CHWs) are not part of a formal system, but they are affiliated with a recognized institution like BRAC NGO in Bangladesh. Restricting it to government affiliation and registration criteria would classify professional CHWs working in NGOs as IPs.
2. IPs have independently (on their own) established their practice and continue to provide health care in the communities they serve. Examples are traditional birth attendants and village doctors in many societies. They provide health care accepted by the community. It includes traditional and religious healing as well as a modern system of medicine.
3. Lastly, they do not possess the recognized certification to provide the kind of care they offer in the community, like traditional birth attendants who lack formal midwifery training.
