# Supplementary material for: Role of informal healthcare providers in tuberculosis care in low- and middle-income countries: A systematic scoping review
Source: PLoS One. 2021 Sep 2;16(9):e0256795. doi: 10.1371/journal.pone.0256795 (PMC8412253; doi:10.1371/journal.pone.0256795)
Supplement: S3 File — (PDF) [file pone.0256795.s003.pdf]

**Table 1: Summary of WHO People-Centered Model of TB Care**

| Type of care | | Functions | Description |
|---|---|---|---|
| **Prevention** | 1 | Health promotion and education | Services include (but are not limited to) awareness-raising and social mobilization activities. |
| | 2 | Immunization | BCG Vaccination |
| | 3 | Latent TB infection screening | |
| | 4 | Latent TB infection prescription | |
| | 5 | Latent TB infection administration | |
| **Detection and diagnosis** | 1 | Active case finding | |
| | 2 | Passive case finding referral | |
| | 3 | Clinical evaluation-TB | |
| | 4 | Lab, X-ray, and others as needed (including sputum sample collection) | |
| **Treatment and support** | 1 | Treatment initiation | Prescription of drugs |
| | 2 | Treatment administration and observation | Administration of drugs (DOTs) |
| | 3 | Monitoring treatment progress and response | Periodic clinical evaluation and lab monitoring |
| | 4 | Prevention and detection of adverse events and comorbidities | |
| | 5 | Diagnosis and treatment of adverse events and comorbidities | |
| | 6 | Treatment lab monitoring | |
| | 7 | Counselling and psychological support | |
| | 8 | Social support | |
| **Total** | **17 functions** | | |

Source: http://www.euro.who.int/__data/assets/pdf_file/0004/342373/TB_Content_WHO_PRO_eng_final.pdf?ua=1
