# Supplementary material for: Role of informal healthcare providers in tuberculosis care in low- and middle-income countries: A systematic scoping review
Source: PLoS One. 2021 Sep 2;16(9):e0256795. doi: 10.1371/journal.pone.0256795 (PMC8412253; doi:10.1371/journal.pone.0256795)
Supplement: S5 File — (PDF) [file pone.0256795.s005.pdf]

Reasons for exclusion of studies:

The reasons for excluding the studies after reading their full texts are listed in Table 1.

Table 1: Characteristics of excluded studies (ordered by publication year)

S. N	Author (Year)	Reason for exclusion
1	Huang et al. (2019)	Lay Health Workers were hired, trained, paid, and involved as part of the care team.
2	Sy T R et al. (2019)	HIV care was provided to the TB patients.
3	Howell et al. (2018)	The paper is focused on HIV care among TB patients.
4	Rogers et al. (2018)	Community Health Workers (CHWs) were trained and deployed by PIH.
5	Kufa et al. (2018)	The focus of the paper is the integration of HIV/TB services in primary health clinics. The TB screening officer was trained, had some prior experience working in healthcare and were involved as part of the project.
6	Masur et al. (2017)	CHWs were part of GHESKIO, which is the largest provider of HIV and TB services in the Caribbean.
7	Brown et al. (2017)	Volunteers were developed by the World Relief as part of their project and were paid by them.
8	Querri et al. (2017)	Community Health Volunteers (CHVs) were part of NGOs involved in the study.
9	McAllister et al. (2017)	CHWs were part of community health centers.
10	Datiko, D. G. et al. (2017)	Cadre of national CHWs.
11	Getnet et al. (2017)	Cadre of national CHWs.
12	Adejumo et al. (2016)	All the models were designed and implemented by different funding and implementing partners. There was a structured hiring, training, and supervision process.
13	Bhardwaj, R. R. et al. (2016)	Both are formally recognized private health practitioners in India.
14	Napier et al. (2016)	Conference abstract.
15	Kaplan et al. (2016)	Lack of clear definition for Community care workers (CCWs).
16	Volkmann et al. (2016)	The program was built on existing national TB program, and CHVs were cadre of national CHWs.
17	Howard et al. (2016)	Study protocol.
18	Flick R J et al. (2016)	Conference abstract.
19	Ritchie et al. (2015)	The LHWs were paid, trained and part of health system. Also, the focus of the study was to evaluate the knowledge translation intervention among LHWs.
20	Adewole et al. (2015)	Providers are formal staff working at TB centers.
21	Balogun et al. (2015)	Providers are community volunteers.

22	Ong'ang'o, J. R et al. (2014)	Cadre of CHWs for Ministry of Health supported by development partners.
23	Viney et al. (2014)	Study was KAP of traditional healers and assessing the feasibility and willingness to involve in the National TB program.
24	Islam et al. (2013)	CHVs were frontline health workers of BRAC.
25	Soares et al. (2013)	Lay health workers were paid employee of a municipality.
26	Crispim et al. (2012)	Cadre of national CHWs.
27	Uwimana et al. (2012)	The Community Care Workers (CCWs) were managed and deployed by NGOs supporting government.
28	Khan, A. J. et al. (2012)	Local residents were involved as screeners.
29	Uwimana et al. (2012)	The CCWs were either deployed by NGO or government.
30	Soomro et al. (2012)	Providers are community volunteers.
31	Mashamba et al. (2011)	Focus of the study is HIV/AIDs.
32	Prado et al. (2011)	CHWs were part of national TB control strategy.
33	Divakaran et al. (2011)	The goal of the study was to assess the availability, sale and magnitude of anti-tuberculosis drugs dispensing through private pharmacies.
34	Mafigiri et al. (2011)	Treatment supporters were either patient's family members or any other relation.
35	Onyeneho et al. (2011)	Qualitative perspective study
36	Datiko et al. (2010)	Cadre of national CHWs and the focus of the paper was cost-effectiveness of the community-based model.
37	Onyeneho et al. (2010)	Exploratory study understanding the willingness and capability of Patent Medicine Vendors (PMVs).
38	Herce et al. (2010)	Cadre of trained CHWs deployed by an NGO.
39	Hall V C et al. (2010)	Focus on mental health.
40	Scatolin et al. (2009)	Cadre of national CHWs.
41	Datiko, D. G. et al. (2009)	Cadre of national CHWs.
42	Simon et al. (2009)	Cadre of national CHWs developed by Ministry of Health.
43	Egwaga et al. (2009)	Treatment was supported by a family member.
44	Gai et al. (2008)	Cadre of trained formal community health workers
45	Ahmadzai et al. (2008)	Cadre of trained CHWs.
46	Awofeso et al. (2008)	Study compares the quality, quantity and distribution of tuberculosis physicians, laboratory staff, community health workers and nurses in Nigeria and Kyrgyzstan.
47	Chimbanrai et al. (2008)	The treatment supporters are not defined, but in the discussion, it is mentioned that for most of the patients, family members acted as a treatment supporter.

48	Phomborphub et al. (2008)	Village Health Volunteers (VHVs) were cadre of national health workers.
49	Rojpibulstit, M. et al. (2007)	Focus on drug store personnel's practises on management of suspected TB cases.
50	Xiong et al. (2007)	Village doctors are national cadre of CHWs and are part of formal system in China.
51	Cavalcante et al. (2006)	CHWs were hired in a structured way, trained, and then deployed.
52	Peltzer et al. (2006)	Focus of the study is HIV/AIDs.
53	Clarke et al. (2006)	Linked publication.
54	Newell, J. N. et al. (2006)	Family members are considered as caregivers.
55	Barker et al. (2006)	This paper does not explore the role of THs in TB care. Focused on examining the direct relationship between visiting traditional healers, increased morbidity, and death from TB.
56	Salim et al. (2006)	Linked publication.
57	Singh, A. A. et al. (2005)	Angan-wari workers (AWs) are national cadre of CHWs.
58	Clarke et al. (2005)	The LHWs were trained using structured training module, were part of the team and had defined role and responsibility. LHWs were farmers.
59	Escott et al. (2005)	The CHWs are developed and deployed by government, and the focus of the paper is to understand the experience of people involved in community-based TB program.
60	Shargie et al. (2005)	Health promoters were trained and paid CHWs.
61	Wright, J. et al. (2004)	Cadre of national CHWs.
62	Kironde et al. (2004)	Objective was to evaluate the role of NGOs in TB treatment using qualitative methods.
63	Lambert et al. (2004)	A survey of pharmacies.
64	Banerjee et al. (2004)	Study assessing acceptability of Traditional Healers (THs) in tuberculosis control program.
65	Dudley et al. (2003)	Treatment supporters were recruited and trained by NGOs supporting community DOTs program.
66	Lwilla, F. et al. (2003)	Providers were community members who worked as volunteers.
67	Kironde et al. (2002)	Community members who are enrolled as volunteers.
68	Islam et al. (2002)	CHWs were developed and employed by BRAC.
69	Khan et al. (2002)	National community health worker program.
70	Pungrassami et al. (2002)	No definition was provided for village health volunteers are and the focus of the study was to compare practice of DOTs among different kind of observers as part of National TB program in Thailand.
71	Rajeswari et al. (2002)	Study was done to assess the willingness of private pharmacists to participate in National TB program.
72	Zwarenstein et al. (2000)	Provider definition was mentioned in previous publication:<br>https://www.ncbi.nlm.nih.gov/pubmed/8796257<br>They were community member working as volunteers.
73	Islam et al. (1999)	CHWs were developed and employed by BRAC.

74	Jones, J. S. et al. (1998)	It is a news article.
75	Jagota et al. (1998)	Linked publication.
76	Dick et al. (1997)	The providers are farmers.
77	Chowdhury et al. (1997)	CHWs were developed and employed by BRAC.
78	Wilkinson et al. (1997)	Voluntary Lay People (VLP) were not defined in the study.
79	Singh, J V et al. (1989)	Conference abstract
