# Supplementary material for: Role of informal healthcare providers in tuberculosis care in low- and middle-income countries: A systematic scoping review
Source: PLoS One. 2021 Sep 2;16(9):e0256795. doi: 10.1371/journal.pone.0256795 (PMC8412253; doi:10.1371/journal.pone.0256795)
Supplement: S6 File — (PDF) [file pone.0256795.s006.pdf]

Quality assessment of included studies:

Table 1: QA of studies with Quasi-experimental design (QED)

S. N	Study	Q1	Q2	Q3	Q4	Q5	Q6	Q7	Q8	Q9	Total # of Yes
1	Sima et al. (2019) (1)	Y	Y	NA	N	N	N	NA	UC	N	2
2	Dutta et al. (2018) (2)	Y	Y	Y	Y	Y	Y	Y	Y	Y	9
3	Colvin et al. (2014) (3)	Y	Y	NA	N	Y	NA	NA	Y	N	4
4	Simwaka et al. (2012) (4)	Y	Y	Y	Y	N	Y	Y	Y	Y	8
5	Kaboru et al. (2013) (5)	Y	Y	NA	N	N	NA	NA	UC	N	2
6	Salim et al. (2006) (6)	Y	Y	NA	Y	N	UC	NA	Y	N	4
7	Harper et al. (2004) (7)	Y	Y	NA	N	N	Y	NA	N	N	3
8	Colvin et al. (2003) (8)	Y	Y	Y	Y	N	UC	Y	Y	N	6
9	Kangangi et al. (2003) (9)	Y	Y	Y	UC	N	UC	Y	Y	Y	6
10	Jagotal et al. (1997) (10)	Y	Y	UC	Y	N	UC	Y	UC	N	4
Total # of Yes		10	10	4	5	2	3	5	6	3	

Y: Yes, N: No, UC: Unclear, NA: Not applicable Q1: Is it clear in the study what is the 'cause' and what is the 'effect' (i.e. there is no confusion about which variable comes first)? Q2: Were the participants included in any comparisons similar? Q3: Were the participants included in any comparisons receiving similar treatment/care, other than the exposure or intervention of interest? Q4: Was there a control group? Q5: Were there multiple measurements of the outcome both pre and post the intervention/exposure? Q6: Was follow up complete and if not, were differences between groups in terms of their follow up adequately described and analyzed? Q7: Were the outcomes of participants included in any comparisons measured in the same way? Q8: Were outcomes measured in a reliable way? Q9: Was appropriate statistical analysis used?

Table 2: QA of studies with Experimental design

S. N	Study	Q1	Q2	Q3	Q4	Q5	Q6	Q7	Q8	Q9	Q10	Q11	Q12	Q13	Total # of yes
11	Bello et al. (2017) (11)	Y	NA	Y	NA	NA	Y	Y	Y	Y	Y	Y	Y	Y	10

Y: Yes, N: No, UC: Unclear, NA: Not applicable Q1: Was true randomization used for assignment of participants to treatment groups? Q2: Was allocation to treatment groups concealed? Q3: Were treatment groups similar at the baseline? Q4: Were participants blind to treatment assignment? Q5: Were those delivering treatment blind to treatment assignment? Q6: Were outcomes assessors blind to treatment assignment? Q7: Were treatment groups treated identically other than the intervention of interest? Q8: Was follow up complete and if not, were differences between groups in terms of their follow up adequately described and analyzed? Q9: Were participants analyzed in the groups to which they were randomized? Q10: Were outcomes measured in the same way for treatment groups? Q11: Were outcomes measured in a reliable way? Q12: Was appropriate statistical analysis used? Q13: Was the trial design appropriate, and any deviations from the standard RCT design (individual randomization, parallel groups) accounted for in the conduct and analysis of the trial?

References:

1. Sima BT, Belachew T, Bjune G, Abebe F. Traditional healers' role in the detection of active tuberculosis cases in a pastoralist community in Ethiopia: a pilot interventional study. BMC Public Health. 2019;19(1):1-7.
2. Dutta A, Pattanaik S, Choudhury R, Nanda P, Sahu S, Panigrahi R, et al. Impact of involvement of non-formal health providers on TB case notification among migrant slum-dwelling populations in Odisha, India. PLOS One. 2018;13(5):e0196067.
3. Colvin C, Mugyabuso J, Munuo G, Lyimo J, Oren E, Mkomwa Z, et al. Evaluation of community-based interventions to improve TB case detection in a rural district of Tanzania. Global Health: Science and Practice. 2014;2(2):219-25.
4. Simwaka BN, Theobald S, Willets A, Salaniponi FM, Nkhonjera P, Bello G, et al. Acceptability and effectiveness of the storekeeper-based TB referral system for TB suspects in sub-districts of Lilongwe in Malawi. PLOS One. 2012;7(9):e39746.
5. Kaboru BB. Active referral: an innovative approach to engaging traditional healthcare providers in TB control in Burkina Faso. Healthcare Policy. 2013;9(2):51.
6. Salim H, Uplekar M, Daru P, Aung M, Declercq E, Lönnroth K. Turning liabilities into resources: informal village doctors and tuberculosis control in Bangladesh. Bulletin of the World Health Organization. 2006;84:479-84.
7. Harper M, Hill P, Bah A, Manneh K, McAdam K, Lienhardt C. Traditional healers participate in tuberculosis control in The Gambia. The International Journal of Tuberculosis and Lung Disease. 2004;8(10):1266-
8. Colvin M, Gumede L, Grimwade K, Maher D, Wilkinson D. Contribution of traditional healers to a rural tuberculosis control programme in Hlabisa, South Africa. The International Journal of Tuberculosis and Lung Disease. 2003;7(9):S86-S91.
9. Kangangi J, Kibuga D, Muli J, Maher D, Billo N, NÕgangÕa L, et al. Decentralisation of tuberculosis treatment from the main hospitals to the peripheral health units and in the community within Machakos district, Kenya. The International Journal of Tuberculosis and Lung Disease. 2003;7(9):S5-S13.
10. Jagota P, Balasangameshwara V, Jayalakshmi M, Islam MM. An alternative method of providing supervised short course chemotherapy in District Tuberculosis Programme. Indian Journal of Tuberculosis. 1997;44:73-8.
11. Bello G, Faragher B, Sanudi L, Namakhoma I, Banda H, Malmborg R, et al. The effect of engaging unpaid informal providers on case detection and treatment initiation rates for TB and HIV in rural Malawi (Triage Plus): A cluster randomised health system intervention trial. PLOS One. 2017;12(9):e0183312.
