# Supplementary material for: Role of informal healthcare providers in tuberculosis care in low- and middle-income countries: A systematic scoping review
Source: PLoS One. 2021 Sep 2;16(9):e0256795. doi: 10.1371/journal.pone.0256795 (PMC8412253; doi:10.1371/journal.pone.0256795)
Supplement: S7 File — (PDF) [file pone.0256795.s007.pdf]

Classification system for IPs based on their practice:

1. **Traditional Health Practitioners (THPs)**: This group includes providers who base their practice on a traditional healing system like spiritual healers, herbalist, bone settlers etc.

2. **Traditional Birth Attendants (TBAs):** This group includes providers who are not formally trained but assist a woman during childbirth in the community. They are also referred to as Dais.

3. **Drug sellers, storekeepers, and chemists:** This group includes providers who own stores and sell medicines in the community. The storekeepers included in the review sold simple medicines like cough remedies to the patients from their grocery store.

4. **Untrained allopathic practitioners:** This group includes providers identified as village doctors in the communities they serve. They practice an allopathic system of medicine but lack formal training in this discipline.
